# SUZY^TM^ forceps facilitate nasogastric tube insertion under McGRATH^TM^ MAC videolaryngoscopic guidance: A randomized, controlled trial

**DOI:** 10.1097/MD.0000000000022545

**Published:** 2020-10-09

**Authors:** Kenta Furutani, Tatsunori Watanabe, Keiichiro Matsuda, Yoshinori Kamiya, Hiroshi Baba

**Affiliations:** a Department of Anesthesiology, Niigata University Medical and Dental Hospital; b Department of Anesthesiology, Uonuma Institute of Community Medicine, Niigata University Medical and Dental Hospital, Minami-Uonuma, Niigata, Japan.

**Keywords:** Magill forceps, McGRATH^TM^ MAC, nasogastric tube, SUZY forceps, videolaryngoscope

## Abstract

**Background:**

Nasogastric tubes can be easily inserted in patients under general anesthesia. However, for difficult cases, insertion techniques that can be used in routine clinical practice are limited. SUZY forceps are designed for the removal of pharyngolaryngeal foreign bodies under guidance of a McGrath videolaryngoscope. We hypothesized that using SUZY forceps under McGrath videolaryngoscopic guidance may facilitate nasogastric tube insertion and tested this in a randomized controlled trial.

**Methods:**

Adult patients who underwent gastrointestinal or hepato-pancreato-biliary surgery were randomly allocated to 2 groups; the SUZY group and the Magill group. Patients, nurses, and all clinical staff except for the attending anesthesiologist were blinded to group assignment throughout the study. After anesthesia induction, insertion of the nasogastric tube was performed by skilled anesthesiologists with either SUZY or Magill forceps according to group allocation under McGrath videolaryngoscopic guidance. The primary endpoint was insertion time which was defined as the time required to advance the nasogastric tube by 55 cm from the nostril. Secondary endpoints were the success rates of the nasogastric tube insertion, which were defined as a 55-cm advancement from the nostril at the 1st, 2nd, and 3rd attempt, proper insertion rate, the severity of pharyngolaryngeal complications, and hemodynamic parameters during nasogastric tube insertion.

**Results:**

Sixty patients were randomized and none of these patients were excluded from the final analysis. The median [interquartile range] insertion time was 25 [18–33] seconds in the SUZY group, and 33 [21–54] seconds in the Magill group (*P* = .02). Success rates were not different between the groups (97% and 80% in the SUZY and Magill group at 1st attempt, respectively, *P* = .10). Both, the severity score of the mucosal injury and the severity of sore throat were higher in the Magill than in the SUZY group, whereas the degree of hoarseness did not differ between the 2 groups. Hemodynamic parameters were not significantly different between the groups.

**Conclusion:**

Using SUZY forceps under McGrath videolaryngoscopic guidance reduced the time required to insert a nasogastric tube and the severity of pharyngolaryngeal complications, when compared to using Magill forceps.

## 1 Introduction

A nasogastric tube (NGT) is often used for the aspiration of gastric contents during general anesthesia. Usually, the insertion of an NGT is an easy procedure when the patient is under general anesthesia. However, insertion techniques that can be used in routine clinical practice for difficult cases are limited. Although various techniques that can facilitate NGT insertion have been reported,^[1–7]^ those techniques are performed blindly, which can occasionally cause serious complications, such as mucosal injury, bleeding, edema, and tracheal displacement.^[8]^ Therefore, whenever possible, anesthesiologist should perform NGT insertion under visualization of the larynx and the pharynx to avoid these complications.

Use of Magill forceps under the guidance of the Macintosh laryngoscope is a popular technique for NGT insertion because it allows both direct visualization of the larynx and insertion of NGT into the esophagus by means of the forceps. However, it is sometimes difficult to visualize the pharynx and the esophageal inlet clearly, particularly when the trachea is intubated. In addition, this technique requires a certain skill level.

Although videolaryngoscopes, such as the Glidescope^TM^ (Verathon, Bothell, WA)^[9–11]^ or Pentax-AWS^TM^ (Hoya, Tokyo, Japan),^[12–15]^ may allow both visualization of the esophageal inlet and facilitation of NGT insertion, they cannot direct the tip of the NGT to the esophageal inlet. In fact, NGT insertion using the King Vision^TM^ (King Systems, Indianapolis, IN) failed to demonstrate significant benefit over blind insertion.^[16]^ Therefore, videolaryngoscopes are not always useful, particularly when the tip of the NGT does not advance to the esophagus. To overcome this limitation, a dedicated device is required to pass through the esophageal inlet for each videolaryngoscope.

**Figure 1:**
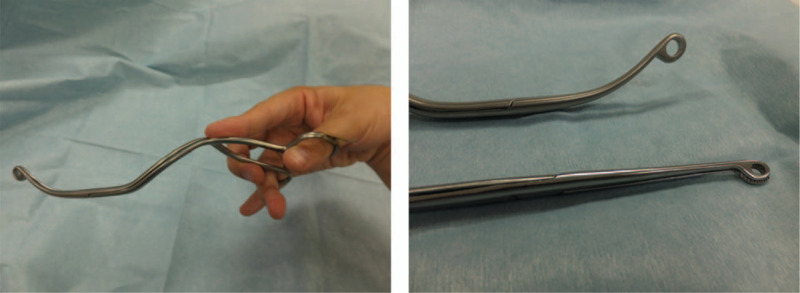
SUZY forceps. The SUZY forceps are curved from the middle to distal portion while the Magill forceps are straight. The curved form of the SUZY forceps fits the blade of the McGrath MAC videolaryngoscope.

SUZY^TM^ forceps (TDM Corporation, Tokyo, Japan) are designed for the removal of pharyngolaryngeal foreign bodies,^[17,18]^ particularly under guidance of the McGRATH^TM^ MAC videolaryngoscope (Aircraft Medical Ltd, Edinburgh, UK). Because the curved form of SUZY forceps fits the blade of the McGrath (Fig. 1), the tips of the forceps can be clearly visualized on the McGrath screen. In a Mannikin study, we reported that SUZY forceps facilitated NGT insertion more effectively than did Magill forceps under McGrath videolaryngoscopic guidance.^[19]^ Moreover, because a recent randomized controlled trial revealed that using the modified Magill forceps with the Glidescope facilitated NGT insertion,^[20]^ it seemed possible that using SUZY forceps with the McGrath videolaryngoscope could also facilitate NGT insertion. However, this has not yet been established in clinical practice.

We hypothesized that using SUZY forceps under McGrath videolaryngoscopic guidance would enable easier NGT insertion because it would allow clear visualization of the esophageal inlet and facilitate directing the NGT tip to the esophagus. To test our hypothesis, we performed a randomized controlled trial in patients who were scheduled to undergo gastro-intestinal or hepato-pancreato-biliary surgery. The aim of the present study was to develop an improved NGT insertion technique in patients under general anesthesia, which would lead to improvements in patient safety. We present our approach to comparing the different forceps and the results of the comparison, and discuss the efficacy and limitations of videolaryngoscopy for NGT insertion.

## 2 Methods

This prospective, randomized, open blinded endpoint (PROBE) study was approved by the Ethics Committee of Niigata University (Niigata, Japan, document No. 2097), and written informed consent was obtained from all subjects participating in the trial before their recruitment into the study. The trial was registered before patient enrolment in UMIN-CTR (registration number: UMIN000016631, Principal investigator: Kenta Furutani, Date of registration: February 25, 2015). The study was conducted at the Niigata University Medical and Dental Hospital (Niigata, Japan) between April and November 2015. This manuscript adheres to the applicable CONSORT guidelines.

### 2.1 Participants

Adult patients who were scheduled to undergo gastro-intestinal or hepato-pancreato-biliary surgery in Niigata University Medical and Dental Hospital between April 2015 and November 2015 were enrolled and assessed for eligibility. The exclusion criteria were the following: age less than 20 years; inability to communicate; preoperative risk factors for pulmonary aspiration of gastric contents; anticipated difficult airway; presence of tracheostomy; pre-existing hypoxia/respiratory failure before surgery; coagulopathy; history of esophageal/gastric varix or esophageal cancer; history of pharyngolaryngeal lesion; history of cardiovascular/cerebrovascular disease; history of emergency surgery; the presence of any fragile teeth; the presence of allergies or contraindications to any of the drugs used in the study; unwillingness to participate.

### 2.2 Randomization

A research assistant used computer-generated block randomization (block size: 30) to allocate 60 eligible, consecutive patients to the SUZY group or the Magill group randomly in a 1:1 ratio (Research Randomizer; https://www.randomizer.org/). The randomization allocation sequence was concealed in sealed, prenumbered, opaque envelopes prepared by the research assistant. Patients, nurses, and all clinical staff, except for the attending anesthesiologist, were blinded to both group assignments and the study endpoints, throughout the study.

### 2.3 Study protocol

After preoxygenation with 5 L/min of oxygen, general anesthesia was induced by bolus administration of propofol with continuous infusion of remifentanil, and was maintained using target-controlled infusion of propofol or inhalation of volatile agents (sevoflurane or desflurane) to maintain a bispectral index (BIS) value between 40 and 60. After anesthesia induction, rocuronium bromide (0.6–0.9 mg/kg) was intravenously administered. The attending anesthesiologist ventilated the patient's lung using a facemask until the researcher confirmed the vanishing of T1 under the train-of-four monitoring (TOF watch; Nihon Kohden, Tokyo, Japan).

Before tracheal intubation, an operator (KF or TW) who was familiar with handling both types of forceps started to insert a 14 Fr-sized NGT (NST-14, NIPRO, Osaka, Japan) from either nostril with either SUZY or Magill forceps (ACOMA Medical Industry, Tokyo, Japan), according to the allocation, under McGrath videolaryngoscopic guidance. The NGT was lubricated using water-soluble jelly (CaineZero jelly, Nagase Medicals, Itami, Japan) before insertion. The blade of the McGrath videolaryngoscope was decided depending on the patient's sex; size 4 for male and size 3 for female.

Insertion of the NGT was performed by skilled anesthesiologists who had experience with more than 20 cases of NGT insertion using SUZY forceps. After the NGT was inserted from the nostril and it was confirmed that the tip of the NGT could be visualized on the screen of the McGrath videolaryngoscope, a nurse blinded to allocation began to measure the insertion time with a stopwatch, while the operator attempted to advance the NGT 55 cm from the nostril. Insertion of the NGT was performed by relying only on the image on the screen of the McGrath videolaryngoscope. Manipulation was performed within the range of the screen and the researcher avoided blind manipulation whenever possible. To avoid prolonged non-ventilation time and desaturation, if NGT insertion did not succeed within 60 seconds, NGT insertion and measuring of insertion time were paused. During the interruption, mask ventilation was performed with 100% oxygen for at least 1 minute. After checking that SpO_2_ had recovered to 100%, NGT insertion and measuring were resumed. Failure to advance the NGT 55 cm from the nostril by the 3rd attempt (insertion time >180 seconds) was defined as “failure,” for safety reasons, and the study was not conducted further. At this point, the insertion time was recorded as 180 seconds. After measurement of the insertion time was finished, mask ventilation was started again, and tracheal intubation was performed using the McGrath videolaryngoscope. At that time, the operator observed the pharynx and evaluated the severity of the mucosal injury using a 4-point scale (0: none, 1: submucosal hemorrhage, 2: mucosal injury with slight hemorrhage, 3: mucosal injury with active bleeding).

The success of NGT insertion was defined as a 55-cm advancement from the nostril. The proper placement was confirmed by aspiration of gastric contents or auscultation of a gurgling sound using a stethoscope after injecting 20 mL of air into the NGT. If insertion of the NGT could not be confirmed by those methods, kinking of the tube in the mouth or pharynx was checked using the McGrath videolaryngoscope. After surgery, insertion of the NGT was finally confirmed by a postoperative abdominal radiograph. On the day after the operation, the degree of hoarseness and sore throat was evaluated using a 4-point scale (0: none, 1: slight, 2: moderate 3: severe) at a postoperative examination performed by the attending anesthesiologist.

### 2.4 Endpoints

The primary endpoint was the insertion time which was defined as the time required to advance the NGT 55 cm from the nostril. Secondary endpoints were the success rates of the NGT insertion at the 1st (<60 seconds), 2nd (<120 seconds), and 3rd attempt (<180 seconds), proper insertion rate, defined as the aspiration of gastric contents or auscultation, the severity of pharyngolaryngeal complications (mucosal injury, hoarseness, and sore throat), and hemodynamic parameters (blood pressure and heart rate) during NGT insertion.

### 2.5 Statistical analysis

The sample size was calculated using G-power 3.1 software (Heinrich Heine, University of Düsseldorf, Düsseldorf, Germany). Before this study, we had conducted a pilot study involving 10 patients (unpublished data). The pilot study showed that the insertion time was 41.8 [14.2] seconds (mean [standard deviation]), when we inserted the NGT using SUZY forceps. We expected that SUZY forceps would reduce the time required to insert the NGT by 10 seconds, as compared to Magill forceps. As a result, a sample size calculation determined that 26 patients per group was the smallest sample size required to demonstrate a difference with a statistical power of 0.8 and a type 1 error rate of 0.05 using an independent samples *t*-test. After considering possible dropouts, 30 patients were enrolled per group.

Data were analyzed with GraphPad Prism 7.0 (GraphPad Software, San Diego, CA) software. We compared the insertion time between groups, using the Mann–Whitney *U* test. Categorical data and the rating scales were analyzed using Fisher exact test or the chi-square test. Parametric data were analyzed using Student *t* test. A *P*-value of less than .05 was considered statistically significant. Data are expressed as median [interquartile range].

## 3 Results

**Figure 2:**
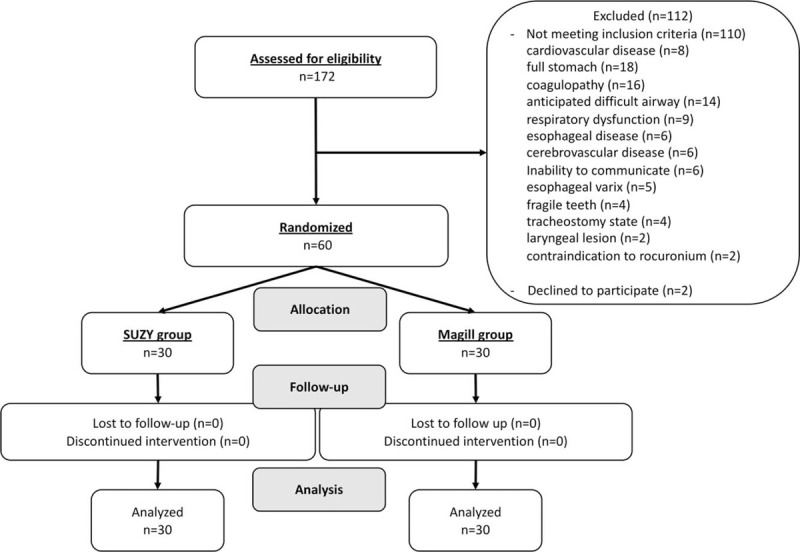
CONSORT flow diagram.

One hundred seventy-two patients were assessed for eligibility, and 112 patients were excluded according to the exclusion criteria. Sixty patients were randomized to the 2 groups, none of whom were excluded from the final analysis (Fig. 2). Data were collected between April 2015 and November 2015. The patients’ characteristics did not differ between the groups (Table 1).

**Table 1:**
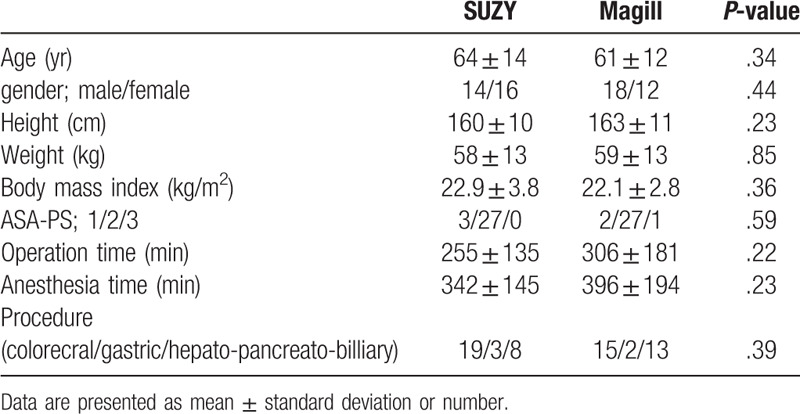
Baseline characteristics and perioperative data of the study participants.

**Figure 3:**
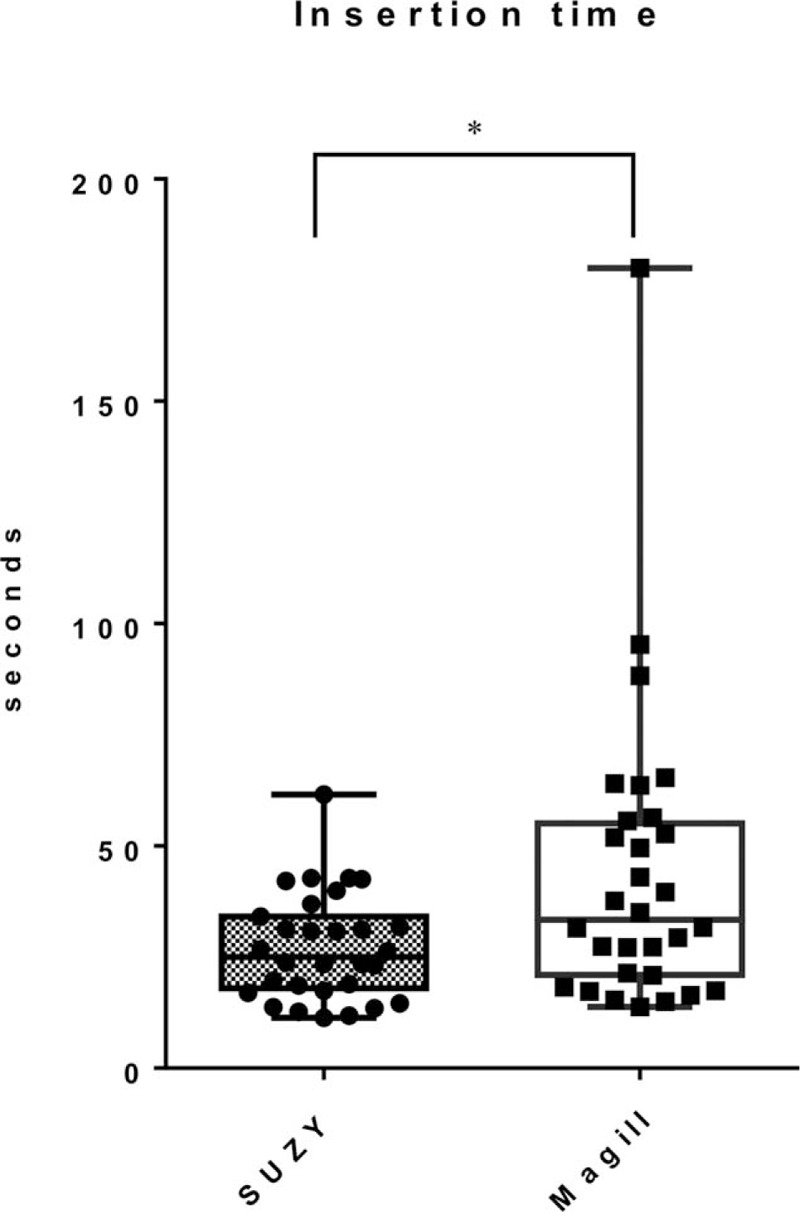
Nasogastric tube insertion time. The bottom and top of the boxes represent the first and third quartiles, and the bands inside the boxes represent the median. The ends of the whiskers represent the maximum and the minimum values. The insertion time (median [interquartile range]) was 25 [18–33] s in the SUZY group and 33 [21–54] s in the Magill group. *P*-values were calculated using the Mann–Whitney *U* test. ^∗^*P* < .05.

The insertion time was significantly shorter in the SUZY group than in the Magill group (25 [18–33] seconds and 33 [21–54] seconds, respectively, *P* = .02, Fig. 3).

Success rates, which were defined as a 55-cm advancement from the nostril at the 1st, 2nd, and 3rd attempt, were not different between the groups although the success rate at the 1st attempt tended to be higher in the SUZY group (97% and 80%, respectively, *P* = .10, Fisher exact test, Table 2A). The success rate at the 3rd attempt was 100% in the SUZY group and 97% in the Magill group. We could not advance the NGT within the 3rd attempt in 1 case in the Magill group. Rates of proper placement of the NGT, as diagnosed by aspiration of gastric contents or auscultation of a gurgling sound after injecting air into the NGT, were not different between the groups (93% in the SUZY group and 90% in the Magill group, *P* > .99, Fisher exact test, Table 2B). We could not confirm the proper placement of the NGT in 5 cases (2 in the SUZY group and 3 in the Magill group). In 4 of these 5 cases, NGT placement could not be confirmed by observation with the McGrath videolaryngoscope or postoperative radiographs. The causes were kinking of the NGTs at the pharynx (2 cases; 1 case per group) and not passing through the lower esophageal sphincter (2 cases; 1 case per group).

**Table 2:**
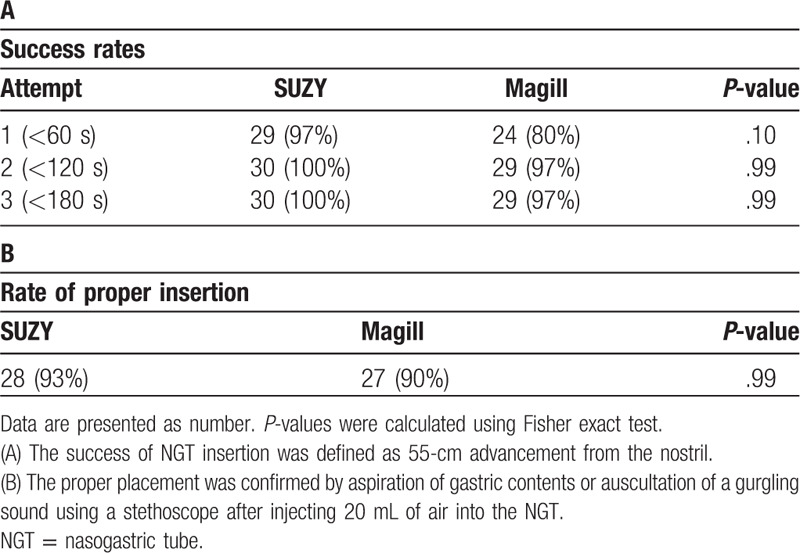
Success rates and rate of proper placement of NGT insertion.

Both the severity score of the mucosal injury and the severity of sore throat was higher in the Magill than in the SUZY group, whereas the degree of hoarseness did not differ between the groups (Table 3). Hemodynamic parameters, blood pressure, and heart rate, before and after NGT insertion were not statistically significantly different between groups.

**Table 3:**
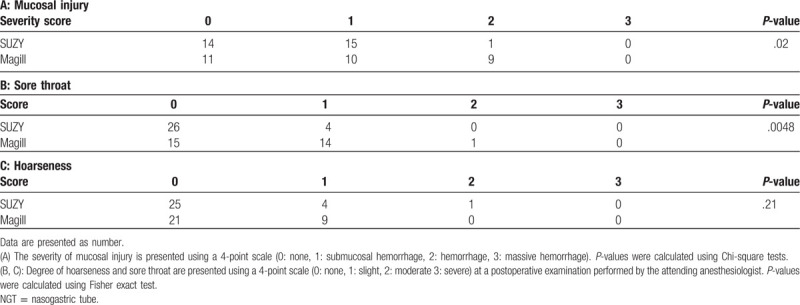
Pharyngolaryngeal complications after insertion of NGT.

## 4 Discussion

We tested whether using SUZY forceps under McGrath videolaryngoscopic guidance could facilitate NGT insertion. In this PROBE study, the SUZY forceps were shown to reduce both the time required for NGT insertion and the severity of pharyngolaryngeal complications, as compared with use of Magill forceps under McGrath videolaryngoscopic guidance.

A previous report indicated that the most common site of NGT impaction was the arytenoid cartilage and piriform sinus, when the NGT entered the hypopharynx.^[21]^ Impaction could be avoided using SUZY forceps combined with McGrath videolaryngoscopic guidance, because the hypopharynx could be easily visualized on the McGrath screen and the NGT could then be advanced to the esophageal inlet by means of the SUZY forceps. The results of the present study indicate that the SUZY forceps can be an effective tool for safe and easy insertion of the NGT.

Our previous manikin study^[19]^ demonstrated that SUZY forceps could advance the NGT for a longer distance than Magill forceps during insertion of the NGT from the pharynx to the esophagus. This advantage likely reduced the insertion time. Although the difference between both groups was small, there is no alternative clinical measure, other than time of insertion, of forceps-performance in NGT insertion. Because a shorter insertion time should lead to a lower incidence of pharyngolaryngeal complications, this difference, although small, was clinically significant and reflected the efficacy of SUZY forceps.

We could not show a statistically significant difference in the success rate of NGT insertion between groups although the rate of success at the 1st attempt tended to be higher in the SUZY group. This may be due to the relatively high success rate of NGT insertion in this study as compared with previous reports: 80% in the Magill group at the 1st attempt. The success rates of NGT insertion in previous studies using a blinded technique were in the range 34% to 77%.^[1,2,4,5,10,20–22]^ It is possible that the success rate was elevated simply due to use of the McGrath videolaryngoscope, regardless of the forceps used, although no randomized trials have been performed using only McGrath videolaryngoscopy as an intervention for NGT insertion except for our study.^[19]^ Because the tips of both forceps can be clearly visualized on the McGrath monitor, McGrath videolaryngoscopy would improve NGT insertion via Magill forceps as well as via SUZY forceps. Indeed, it has been reported that insertion of a transesophageal echocardiographic probe was facilitated simply by using McGrath videolaryngoscopic guidance.^[23]^ If only the difficult cases were included, it is likely that a difference in the success rate could be found.

Because videolaryngoscopes have a narrower field of view than the Macintosh laryngoscope, we were not able to recognize kinking of the NGT when the NGT advanced into the anterior larynx, which was outside the field of view on the McGrath monitor. Indeed, in the present study, kinking of the NGT was not recognized in 2 cases during NGT insertion. Although videolaryngoscopes facilitate visualization of the larynx, they have limitations when used for NGT insertion.

The present study was performed without tracheal intubation, which would have made NGT insertion difficult. Therefore, as anesthesiologists, we would be interested in determining whether use of the SUZY forceps with the McGrath could overcome this issue. It is also unclear whether the SUZY forceps would be useful when the manipulations are performed by a novice practitioner. Further studies are necessary to clarify these questions.

### 4.1 Generalizability

Because NGT insertion was performed by skilled anesthesiologists in this study, the results cannot be simply applied to anesthesia trainees or residents. However, in our previous manikin study,^[19]^ we showed that the skill required to insert an NGT using Magill forceps would be greater than when using SUZY forceps under McGrath videolaryngoscopic guidance. Therefore, if trainees or residents were to insert the NGT using either of these forceps, the difference may be larger than that found in our present study. In such a case, SUZY forceps would be advantageous, regardless of the level of clinical experience.

### 4.2 Limitations

The present study had some limitations. First, we could not blind the attending anesthesiologists and the operators who inserted the NGT to the group allocation. In addition, even if the nurses who measured the insertion time were blinded to the group allocation, they could see which forceps were used for NGT insertion. However, we thought that the fact that those nurses could see the forceps did not affect the measurement because their task was simply to measure the time using a stopwatch. Second, the present study was performed under general anesthesia without tracheal intubation. The presence of an endotracheal tube would make NGT insertion difficult because both the visualization and the manipulation of both types of forceps was difficult, due to the limited oropharyngeal space after tracheal intubation. Further studies would be required to clarify the influence of tracheal intubation. Third, although videolaryngoscopes or the Macintosh laryngoscope can improve the glottic view and enable us to insert the NGT from the pharynx to the esophagus, they cannot overcome a situation where the NGT cannot pass through the lower esophageal sphincter. Therefore, even if we could avoid blind manipulation by using those laryngoscopes, our results could not guarantee proper placement of NGT in the stomach. Fourth, the evaluation of pharyngolaryngeal complications was not blinded, because the operator performed the evaluation. Although these were not the primary endpoints of the present study, there was a possibility of bias.

In conclusion, use of SUZY forceps enabled anesthesiologists to reduce the time required to insert the NGT and the incidence of pharyngolaryngeal complications, as compared to using Magill forceps under McGrath videolaryngoscopic guidance.

## Acknowledgment

The authors would like to thank Editage for providing editorial assistance.

## Author contributions

Kenta Furutani was the chief investigator and responsible for the organization and coordination of the trial. Kenta Furutani, Tatsunori Watanabe and Keiichiro Matsuda contributed to the study conception, and they also performed material preparation, data collection, and analysis. Kenta Furutani wrote and editted the first draft of the manuscript. Tatsunori Watanabe and Yoshinori Kamiya contributed to the data analysis and the writing of the final manuscript. Hiroshi Baba supervised the project.

## Abbreviations

Abbreviation: NGT = nasogastric tube.
